# Which Aspects of Food Value Promote Consumer Purchase Intent after a Disaster? A Case Study of Salmon Products in Disaster-Affected Areas of the Great East Japan Earthquake

**DOI:** 10.3390/foods8010014

**Published:** 2019-01-04

**Authors:** Takashi Suzuki, Taro Oishi, Hisashi Kurokura, Nobuyuki Yagi

**Affiliations:** 1 Graduate School of Agricultural and Life Sciences, The University of Tokyo, Bunkyo, Tokyo 113-8657, Japan; akrkrh@outlook.jp (H.K.); yagi@fs.a.u-tokyo.ac.jp (N.Y.); 2 School of Marine Life Science, Tokyo University of Marine Science and Technology, 4-5-7, Konan, Minato-ku, Tokyo 108-8477, Japan; toishi0@kaiyodai.ac.jp

**Keywords:** the Great East Japan earthquake, Fukushima nuclear accident, consumer purchase intent, Miyagi, salmon, food value, marketing strategy, factor analysis, structural equation modeling

## Abstract

This research examined post-disaster consumer perception of food value and their effects on purchase intent by focusing on Japanese seafood industry after the Great East Japan earthquake. Online surveys on consumers living in Tokyo and Osaka Prefectures were conducted to investigate consumer value perceptions of Miyagi salmon in 2012 and 2015. Multiple-group structural equation modeling (SEM) on the 2012 survey results showed that desire to contribute to restoration (social value) had the greatest positive influence on purchase intent in both regions. Concern about radiation threats (safety value) had a negative influence on purchase intent, with a stronger impact in Osaka than Tokyo. In comparison, the 2015 results revealed a reduction in the effects of these two potent factors (i.e., safety value and social value) on purchase intent only in Osaka. The beneficial value of seafood had a general positive influence on purchase intent, but its magnitude of effect differed by regional and chronological context. Among these three values, sales promotion with emphasis on social value is more effective than with other values. In cases of future disasters in a similar context, marketers are recommended to adopt different value transfer strategies according to geographical and temporal diversity.

## 1. Introduction

Food value has a distinctive impact on consumer food choice, which has been proposed to be subject to various social factors [1,2]. Disaster is one such influential factor. Modern-day disasters are no longer limited to natural disasters such as hurricanes, earthquakes, and tsunamis. Technological hazards are also considered an environmental problem, and both natural and technological disasters may affect consumer choice of food produced in disaster-affected areas [3,4]. However, researchers have not yet determined which aspects of food value motivate consumer purchase of food produced in regions affected by natural and technological disasters.

The 2011 Great East Japan earthquake was a large-scale disaster which involved both natural and technological hazards. The March 2011 earthquake and tsunami affected many fishery areas in Northeast Japan, in particular the Sanriku coastal area, which is the country’s major fishery and production site. Furthermore, the devastating disaster was followed by the Fukushima Daiichi nuclear power accident, a nuclear disaster rated at Level 7 by the International Nuclear and Radiological Event Scale (INES) and the second of its kind after the 1986 Chernobyl nuclear power accident in the Soviet Union. The disaster caused radioactive gas leakage and groundwater contamination [5]. Fishery products were tested for possible radioactive contamination and were banned from shipping when the radiation level exceeded the government standard [6]. Despite these surveillance measures and the absence of sound scientific evidence for the health risk of seafood from the affected regions, the disaster still damaged the reputation of the seafood products, with persisting reluctance among consumers and retailers [7,8,9].

Then, what are the values of food that will affect consumer purchase intent for seafood produced in disaster-affected areas? The 2011 disaster provoked much research interest in consumer food safety perceptions and attitudes towards food produced in disaster-affected areas [10,11,12]. On the other hand, there is limited research on consumer purchase behavior motivated by willingness to contribute to post-disaster restoration. One recent study examined Japanese consumers’ perceptions of food and durable products produced in areas around the Fukushima nuclear plant. The study findings suggested that consumers’ reactions were mixed, i.e., some reduced purchases while others increased purchases, and that this purchase increase was motivated by consumers’ willingness to contribute to restoration [13]. In fact, the April 2011 sales figures of stores in Tokyo selling regional products from the disaster-hit areas of the prefectures of Iwate, Miyagi, and Fukushima more than doubled in compared with the same time the previous year [14]. Therefore, it seems that food products gained new social value [15] from post-disaster restoration, which enhances consumer purchase intent.

Many studies have been conducted on the value of foods involved in consumers’ purchase decision making [15,16,17]. Lusk and Briggeman [15] summarized values of food in 11 items, and mentioned that these values could be classified into two major groups of personal value and social value. Among them, the value related to the safety of food was included in personal value as a promoter of purchasing. However, as mentioned above, consumers’ concern about food safety is expected to be suppressor of food purchasing after the Great East Japan earthquake [10,11,12]. Therefore, based on these previous works, food values can be broadly reclassified into three major categories: (1) taste, freshness, and nutritional value, which have a huge influence on consumer decision-making in food purchases; (2) value related to food safety; and (3) social value associated with consumers’ willingness to perform a certain social activity, e.g., Japanese consumers’ intended purchasing behavior to contribute to the restoration of disaster-affected areas. These values were considered to have influenced consumer decision-making in food purchases after the disaster. After the disaster, seafood marketers in the disaster-affected areas reported restoring sales channels as their greatest challenge [18], and that they required effective marketing strategies to offer consumer incentives to boost sales [19]. However, it remains unknown how much of an influence each of these values had on consumer purchase intent after the complex hazards (i.e., the complex combination of both natural and technological causes of damage). Several efforts have been made to rebuild sales channels by restoring consumer confidence in the seafood industry, other measures including providing research-based information on the potential radioactive contamination, and taking steps to monitor and restrict the distribution of contaminated products [20,21]. We wondered whether communicating safety value of food products in the disaster-affected area would help promote consumer purchase intent.

Furthermore, it is generally accepted that the environmental risk of an accident recedes as time elapses, and that those farther away from the nuclear power plant tend to be less subject to the nuclear threats. However, consumer concerns about food safety did not necessarily decrease as a result of increased physical distance from the disaster-affected area and/or temporal distance from disaster occurrence. In support of this point, Frank and Schvaneveldt [13] discovered a more salient purchase reduction in relatively distant markets, supposedly less prone to disaster-induced influence. Thus, is the promoting effect of the food value which leads to consumers’ willingness to support the disaster-affected area reduced in areas distant from the disaster area?

Moreover, the literature on post-disaster consumer behavior reveals a need for longitudinal research to determine whether the tendency of consumer food safety concerns will shift with the lapse of time after the disaster [13,22]. This research niche is particularly relevant to the complex 2011 disaster, as the disaster-affected areas have a long road to recovery to repair the damage and eradicate the pollution caused by the nuclear accident. Longitudinal studies along this line would provide necessary information on the aspect of food value to be prioritized to promote purchase intent efficiently, and to devise effective sales strategies for each stage during post-disaster recovery.

This research aims to determine the degree of impact food values had on consumer intent in purchasing food products from areas affected by natural and technological disasters. It also intends to assess the spatial pattern and temporal change by analyzing existing cases of consumer concern after the Great East Japan earthquake. The research findings would provide useful information for food marketers to formulate a recovery plan for sales channel restoration in preparation for similar disasters in the future. Seafood was targeted in this study because fishery was one of the major industries in the disaster-affected areas, and thus, implications from this case study might be applicable to other sales activities in similar disasters in the future. This rest of the paper is organized as follows. We first review previous research on food values and create a conceptual model for investigating the relationship between food values and consumer purchase intent. We then generate hypotheses on whether these values might affect purchase intent differently as the spatial distance from the disaster-affected area increases, and test these hypotheses with the data obtained from an internet survey targeting consumers in Tokyo and Osaka in 2012 (i.e., one year after the disaster) and 2015 (i.e., four years after the disaster).

### 1.1. Conceptual Model

Purchase intent can be examined from the perspective of food values in which consumers have interest when they make purchasing decisions. This research draws from Lusk and Briggeman’s [15] taxonomy of food values: personal values (naturalness, taste, price, safety, convenience, and nutrition) and social values (tradition, origin, fairness, appearance, and environmental impact). Their survey identified safety, one constituent of personal value as the most important food value. The organic food they focused on was preferred by consumers mainly for its safety value in the sense of being free from pesticide in comparison with conventional agricultural foods. Furthermore, consumers’ interest in healthy food has been increasing with prevalence of risk of obesity and its associated illnesses; however, the choice of healthy food is affected by personal and social values with respect to foods [23,24,25]. Many of these values are consistent with the characteristics of seafood pointed out by Carlucci et al. [26] as the promoting factors for consumption. Desire to purchase seafood has increased worldwide and one of the promoting factors of this trend is health benefits [27]. However, there are discussions that consumers also have a perception of potential risks (e.g., contamination caused by micro-organisms, algae bio toxins and chemicals) of seafood consumption, and their perceptions affect seafood purchasing behavior [28,29,30].

According to the survey on Japanese consumers’ concern of food in general conducted by Sugimoto et al. [11], almost half of Japanese consumers had been concerned about the effects of radioactive contamination and overall food safety since the 2011 disaster. They revealed that this trend was salient in specific groups of consumers, such as women and consumers who were married and raising children. Aruga [12] found that Japanese consumers concerned about of agricultural and livestock product safety believed the risk of radioactive contamination after the Fukushima nuclear disaster was higher than ever before. A small number of studies have reported consumer concerns about the seafood produced in the disaster-affected areas. Suzuki and Yagi [8] investigated consumer concerns about the safety of seafood from the area affected by the 2011 disaster including Fukushima found that about 50% of the consumers living in Tokyo and Osaka were concerned about safety of Fukushima’s seafood products, and about 40% were concerned about radioactive contamination of seafood produced in Miyagi, Iwate, and Ibaraki, which were the main disaster-affected prefectures. In 2012, Sakai et al. [9] investigated consumer preferences for salted salmon products, and found that the consumers living in Osaka, relatively far from the disaster-affected area, prefer farmed coho salmon produced in Miyagi (in the northeast of Fukushima) less than that of Tokyo, a neighboring market of the affected area. Although this finding might have been attributed to some factors other than radioactive contamination, they compared the consumers’ willingness to pay in two different scenarios: one involved sampling inspection for radioactive contaminants in each salmon’s lot, which was the most common testing method used in post-disaster inspection; the other adopted complete inspection. The results showed that despite an increase in price of the product due to extra cost, consumers expressed willingness to buy farmed salmon which was supposed to have undergone full inspection. These studies have consistently shown that consumers were more concerned about safety of the seafood produced in Fukushima and its vicinity areas affected by the disaster than that produced in other production sites. Therefore, among the set of personal values listed by Lusk and Briggeman [15], the safety value was considered to be an value with a negative impact on purchase intent in the case of seafood produced in a disaster-affected area, while other personal values (e.g., taste and nutritional values) were assumed to have a positive impact on consumer purchase intent. Thus, we extracted from the list of personal values [15] ones which were related to consumer concerns about the radioactive contamination and created a single category of safety value.

We then considered the social value of seafood produced in disaster-affected areas. After the 2011 disaster, few studies reported an increase in consumer purchase intent of products produced in the disaster-affected areas. Frank and Schvaneveldt [13] conducted consumer surveys in Japan and the United States on fictitious fast food and mobile phones which were supposed to have been produced in Fukushima Prefecture. They reported that instead of an understandable decrease in purchase intent due to health concerns, Japanese consumers stated higher willingness to purchase the products as a financial support to the disaster-affected area. This rather unanticipated consumer behavior was explained by Self-Categorization Theory (SCT) [31] and Collective Resilience Theory (CRT) [32]. According to SCT, people have both personal and collective identities, the balance between which relies heavily on the social context [31,33]. Furthermore, SCT specifies that collective identity can expand to affect others, hence the division between psychological in-group and out-group. People tend to help and provide benefit for their in-group over out-group members. Lantz and Loeb [34] explained that when the consumers fully recognized their social identity as members of a regional group, this identity would add to their willingness to support their own group, and consequently, would promote their preference for the regional products. Derived from SCT and Campbell [35], CRT was established to explain human behavior after the 2005 London Bombings. The theory postulates that shared identity and mutual help are encouraged when people recognize that their common fate is in danger, e.g., threatened by a disaster. Therefore, Frank and Schvaneveldt [13] concluded that despite facing the risk of purchasing radiation-contaminated food, Japanese consumers had a shared identity (e.g., members of the same psychological in-group) with people in the disaster-affected area, thus encouraging purchases. Moreover, Aruga [12] proposed that consumers’ altruistic concern, including environmental concern, was also a promoting factor for purchasing foods from the affected area. Therefore, social value as contextualized in the current study (i.e., that of seafood products from disaster-affected areas) is defined as the value which led consumers to contribute to the restoration of the area by purchasing the seafood. Thus, it was likely that this aspect of food value would promote consumer purchase intent of seafood produced in the disaster-affected area.

On the other hand, as the safety value was singled out, other personal values (e.g., taste and nutritional value) were collectively classified as a category of beneficial value. Previous research suggested that consumer perception of these values was less likely to be affected by the disaster context, therefore they were assumed to have a positive impact on purchase intent as in usual consumption. Thus, three independent categories of values were created, i.e., safety value, social value, and beneficial value.

**Figure 1:**
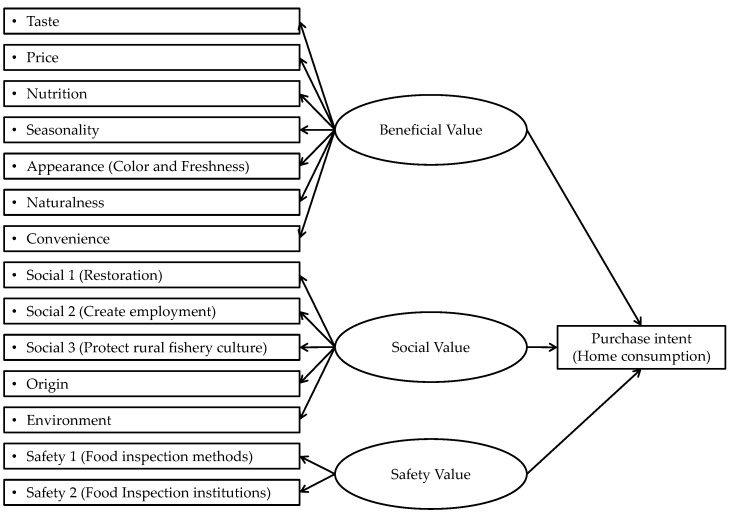
Conceptual model of the relationship between values of seafood and purchase intent. Note: Observable variables of consumer food value perceptions are represented by boxes, and latent variables which exert indirect effects on purchase intent by ellipses.

Figure 1 illustrates the relationship between values of seafood and consumer purchase intent. Overall, purchase intent is influenced by the safety value, beneficial value, and social value perceived in seafood products. These three categories of values serve as latent variables which promote or restrain purchase intent and influence consumer perception of the observable variables of seafood values. In the current study, the role of distance in the magnitude of effect exerted by the three values on purchase intent is examined.

### 1.2. Hypotheses

#### 1.2.1. Social Value

As above mentioned, consumer perception of social value relies on social identity and solidarity with people in disaster-affected areas. In a comparative study on purchase intent of food and non-food products from disaster-affected areas, Frank and Schvaneveldt [13] found U.S. consumers had a greater decrease in purchase intent as compared to Japanese (Tokyo) consumers. However, this cross-regional comparison has not yet been conducted within Japan, especially with a focus on the connection between purchase intent and social value. Frank and Schvaneveldt [22] argued that the consumer purchase increase was motivated by their previous disaster experience. In this light, consumers living in Tokyo were more likely to have in-group favoritism toward people in disaster-affected areas than consumers living in Osaka in the 2011 disaster case, since Tokyo in Eastern Japan suffered more in the disaster than Osaka in the west. Thus, we formulate the following hypothesis on social value and purchase intent.

**Hypothesis 1** **(H1).** *Consumer perception of social value was a greater motivator for purchase intent in Tokyo than in Osaka.*

#### 1.2.2. Safety Value

Previous studies have revealed that consumers living far away from the disaster area had serious concerns about radioactive contamination of food and expressed decreased purchase intent. In a choice-based conjoint analysis of consumer perception of radioactive contamination of agricultural food products, Ujiie [10] discovered that consumers in Osaka showed equally great concern about safety of food from Fukushima Prefecture and its vicinity, despite their little knowledge of food produced near the disabled facility. Similarly, Aruga [12] reported that consumers living in distant areas from the Fukushima nuclear plant tended to believe the existence of a higher risk of radioactive contamination than before the Fukushima nuclear accident and would accept the suspicious food products only with a large discount. These studies indicated that consumers who lived far from the power plant showed greater purchase intent reduction than consumers in neighboring areas. Therefore, we formulate the following hypothesis on regional difference in the magnitude of effect between safety value and purchase intent:

**Hypothesis 2** **(H2).** *Consumer perception of safety value was a greater suppressor for purchase intent in Osaka than in Tokyo.*

#### 1.2.3. Beneficial Value

Previous studies have commonly stated that taste, freshness, nutrition, etc. affect consumers’ choice of food [15,16]. Unlike the safety value and the social value, the beneficial value is assumed to be less susceptible to the context associated with the disaster. Thus, we formulate the following hypothesis on beneficial value and purchase intent:

**Hypothesis 3** **(H3).** *The effect of beneficial value on purchase intent would not differ from one region to the other.*

## 2. Materials and Methods

### 2.1. Target Seafood Product

**Figure 2:**
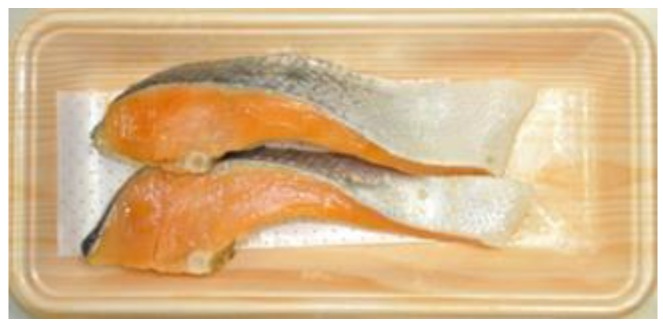
Photo of a Miyagi salmon product.

We chose salted salmon products made from farmed coho salmon (*Oncorhynchus kisutsh*) harvested in Miyagi Prefecture (hereinafter Miyagi salmon) because salted salmon products are one of the most familiar seafood products in Japan and thus would easily evoke the product image in the consumer’s mind. We further ensured the image by presenting a product photo (Figure 2) to the survey participants. Miyagi Prefecture neighbors Fukushima Prefecture to the north, and is Japanese’s largest domestic producer of farmed coho salmon, contributing to 90% of national production volume. The fish holds a special position as a key product in the prefecture’s fisheries promotion policy [36]. However, all the local coho salmon farming facilities were destroyed in the 2011 earthquake and tsunami, causing a drastic decrease in the number of salmon farms [37].

In terms of radioactive contamination, so far no Miyagi salmon has been detected containing a higher level of radioactive contaminants than national standard [38]. However, although Miyagi salmon products have had potentially no harmful effects on consumers’ health, ex-vessel price reduction was reported after the nuclear accident. On this point, Sakai et al. [9] mentioned several possible reasons, among which nuclear disaster was one potent factor. Furthermore, salted salmon made from Miyagi salmon was considered an adequate product for the research because it showcases multiple aspects of the value of seafood which consumers can easily think of.

### 2.2. Questionnaire

As Figure 1 shows, we built a conceptual model connecting consumer purchase intent of Miyagi salmon with the product’s beneficial value, safety value, and social value.

In accordance with the attributes of the target product in the current study, several questions on food values were added to the 2012 questionnaire. Firstly, we specified beneficial value with seven items covering taste, price, appearance, nutrition, seasonality, naturalness and convenience. Value of appearance was defined as color and freshness of the product [26]. We included seasonality as a beneficial value because Japanese consumers tend to believe tasting seasonal seafood is a real pleasure, thus an important value of seafood [39]. Although farming of Miyagi salmon does not use antibiotics in the sea, consumers tend to prefer the certified salmon products which meet regional environmental criteria [40]. Therefore, in this study, value of naturalness was defined as consumers concern about the usage of antibiotics at the salmon farm.

Secondly, we incorporated items on inspection activities in the sub-scale of safety value. It was reported that to deal with the potential radioactive contamination, radiation tests have been conducted by government research institutions and fishery cooperatives, as well as by individual supermarkets and retailers. Test results were displayed in stores, drawing consumers’ attention to food inspection methods, i.e., sampling inspection or complete (100%) inspection, and institutions in charge of inspection.

Thirdly, consumers’ willingness to contribute to restoration of disaster-affected areas by purchasing seafood was taken up as a major attribute of social value of the product. That is, making contributions to restoration not only satisfies a personal desire but also has its social significance (e.g., fairness and tradition). Therefore, three items were added measuring consumers’ perceived social value of Miyagi salmon. Specifically, consumers were asked about their beliefs in consuming the product as a contribution to restoration, creating job opportunities, and protecting rural fishery culture in the disaster-affected areas. Moreover, another item was added evaluating consumers’ intention to purchase Miyagi salmon for home consumption.

As such, we created 15-item questionnaire (Table 1). Consumers’ responses were measured using a five-point Likert scale, ranging from 1 (strongly disagree) to 5 (strongly agree).

Based on the results of 2012 survey and analysis, we established the 2015 questionnaire. The result of factor analysis for the 2012 data, shown in following Section 3.1, suggested some food values were not related to beneficial value, safety value, and social value. Thus, those items with asterisk in Table 1 were excluded from the 2015 questionnaire.

### 2.3. Data

**Figure 3:**
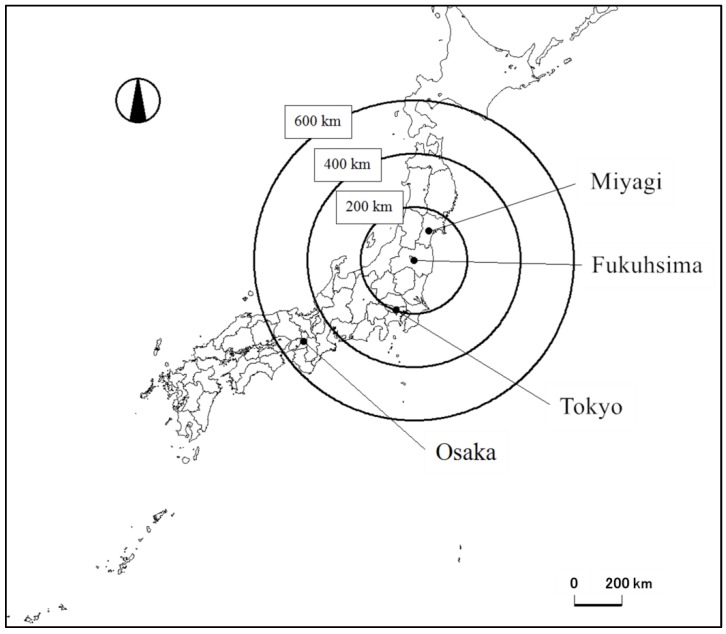
A radius map around the Fukushima nuclear plant. Note: Solid lines indicate borders between prefectures. Black circles indicate radius distances of 200 km, 400 km, and 600 km from Fukushima where the nuclear plant is located.

Online surveys were carried out by Macromil, a survey company in Japan under contract with the University of Tokyo during 21–23 August 2012, and 19–22 March 2015. The target population was consumers aged between 20 and 69 in Tokyo and Osaka. The two cities are respectively about 200 km and 600 km from Fukushima Prefecture, and the largest urban centers in the eastern and western part of Japan. Figure 3 shows the geographical location of Tokyo, Osaka, Miyagi, and Fukushima Prefectures.

We followed the screening processes in a previous study [41] and gave full consideration to the variable of gender (male, female), location (Tokyo, Osaka), and age (20–29, 30–39, 40–49, 50–59, and 60 and above) in drawing a suitable sample from the online survey responses. For a balanced representation, each year sub-groups were created with responses from each category. This resulted in a total of 840 responses in 2012 and 1194 responses in 2015. To further reduce the potential response bias in internet surveys, respondents aged 70 and older were removed, leaving a total of 1853 responses available for analysis [42,43]. These consisted of responses from 659 individuals (*N*_Tokyo_ = 340; *N*_Osaka_ = 319) for 2012 and 1194 individuals (*N*_Tokyo_ = 722; *N*_Osaka_ = 472) for 2015 (hereinafter “the four groups”).

### 2.4. Analysis

To determine the impact of aspects of food value on consumer purchase intent, factor analysis and structural equation modeling (SEM) were conducted. SPSS Version 21.0 and SPSS Amos Version 20.0 statistical software package (IBM, New York, NY, USA) were used for these analyses, respectively.

Prior to factor analysis, Kaiser–Meyer–Olkin (KMO) and Bartlett’s test of sphericity (BTS) were conducted to assess factorability of the data. The value of the KMO ranges from 0 to 1, and a minimum score of 0.60 and a BTS with the significant *p*-value at an alpha level of 0.05 is required for an appropriate analysis [44].

Then, factor analyses were performed on the data of the four groups separately. The purpose of the analysis was to extract construct (e.g., beneficial value, safety value, and social value) by using the observable variables which directly reflected consumer perception of the value of Miyagi salmon. The factor extraction was conducted using the maximum likelihood method, and PROMAX rotation with Kaiser Normalization. Items with strong factor loadings (0.4 or above) were included to form part of a latent variable [45]. Reliability of the latent variables was tested by calculating Cronbach’s alpha, which takes a value between 0 and 1.0, with a recommended threshold level of 0.7 [46]. Then, correlations among three latent variables were checked and added to the conceptual model. To clarify the result of factor analysis, the current study conducted factor analysis based on a polychoric correlation matrix using statistical software R.

To test the fitness of the conceptual model, SEM was carried out separately for each of the four groups. Purchase intent was set as the explained variable and the latent factors of consumer perception of values of Miyagi salmon were set as the explanatory variables. The influence of each latent factor on purchase intent was calculated by standardized path coefficient (β) with the maximum likelihood method. This was to test the validity of the conceptual model for each of the four groups, and to assess regional differences in three hypotheses by comparing the path coefficients.

Next, multiple-group SEM was conducted for the four groups to assess the regional differences of standardized path coefficients so as to accept or reject the hypotheses. Cross-regional differences were assessed using z-tests. Model appropriateness with the data was evaluated by calculating the goodness-of-fit index (GFI), the adjusted goodness-of-fit index (AGFI) and the root mean square error of approximation (RMSEA). GFI and AGFI range from 0 to 1.0, with recommended values close to 1.0. RMSEA also range from 0 to 1.0, with recommended values lower than 0.08 [44].

## 3. Results

### 3.1. Results of Factor Analysis

Table 2 and Table 3 (Note: *df* = degree of freedom; items with factor loadings above 0.4 are presented in boxes. KMO = Kaiser–Meyer–Olkin) present the results of KMO, BTS, and factor analysis. The *p*-values attained in the BTS were significant and the scores of KMO were above the recommended 0.6 in all of the four groups, ensuring a high likelihood of successful factorability of data.

As above mentioned, some food values, i.e., naturalness, convenience, origin and environment showed lower than the threshold of factor loadings (i.e., 0.4 or above) for the 2012 data. These values were excluded from the 2015 survey. Ultimately, three factors were extracted by the Kaiser–Guttmann retention criterion. Beneficial value is related to five items concerning the values of taste, price, nutrition, seasonality, and appearance. Social value is related to three items on how the purchase of Miyagi salmon would contribute to restoration, job creation, and preservation of traditional culture in disaster-affected areas. Safety value is related to two items regarding research institutions in charge of radioactive tests of Miyagi salmon and their inspection methods (complete or sampling inspection). These results of the above mentioned factor analysis were consistent with the results of the analysis based on a polychoric correlation matrix. Cronbach’s alpha values for each of the constructs were over 0.70 for the four groups, indicating a high level of reliability.

**Figure 4:**
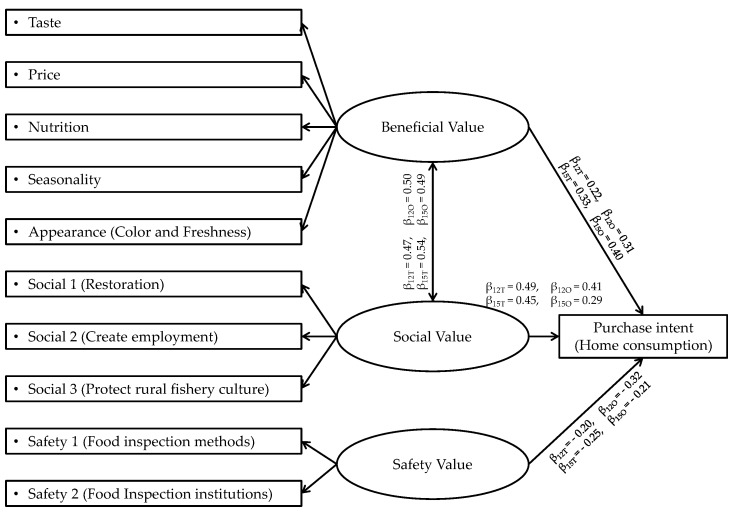
Revised conceptual model of the relationship between values of seafood and purchase intent. Note: β figures correspond to path coefficients of Tokyo 2012 (β_12T_), Osaka 2012 (β_12O_), Tokyo 2015 (β_15T_) and Osaka 2015 (β_15O_). Note: GFI = goodness-of-fit index; AGFI = adjusted goodness-of-fit index; RMSEA = root mean square error approximation; SEM = structural equation modeling.

Results of factor correlation analysis are also displayed in Table 2 and Table 3. The correlation coefficient between beneficial value and social value was above 0.45, large enough to be included in the conceptual model. Thus, conceptual model was revised as shown in Figure 4 based on the results of the factor analysis.

### 3.2. Results of Structural Equation Modeling

SEM was conducted to test whether the model shown in adequately fitted the data of the four groups. Table 4 displays the goodness of fit indices for each group. The results indicated that the conceptual model fitted adequately with the data. The GFI and AGFI, values were close to 1.0, and the RMSEA value was lower than the 0.08 threshold, suggesting a good fit index.

Multiple-group SEM was conducted for the four groups to calculate the standardized path coefficients between each construct and purchase intent, and the effect of regional difference (Note: GFI = goodness-of-fit index; AGFI = adjusted goodness-of-fit index; RMSEA = root mean square error approximation; SEM = structural equation modeling Table 5). The goodness of fit indices indicate that the conceptual model fitted the data satisfactorily well for each of the four groups. Path coefficients between purchase intent and beneficial value, safety value, and social value, and the correlation between beneficial value and social value were significant at the 0.001 level. As predicted, the path coefficient between purchase intent and safety value was negative, and the others were positive.

The 2012 survey results revealed that the most influencial food value on purchase intent was social value (β_Tokyo_ = 0.49; β_Osaka_ = 0.40) in both groups. In Tokyo, the path coefficient between purchase intent and beneficial value (β = 0.22) was higher than that of safety value (β = −0.20). In Osaka, the path coefficient between purchase intent and safety value (β = −0.32) was higher than that of beneficial value (β = 0.31). In both groups, negative effects of safety value and positive effect of beneficial value on purchase intent were almost the same. Z-test results revealed a significant regional difference in negative path coefficients between purchase intent and safety value and it was significantly larger in Osaka than that of Tokyo (z = −2.472, *p* < 0.01). On the other hand, no significant regional difference was found in the positive effects of social value. Therefore, H1 was rejected and H2 was accepted in the 2012 survey. Furthermore, there is no significant regional difference of the path coefficient between purchase intent and beneficial value (β_Tokyo_ = 0.22; β_Osaka_ = 0.31). Thus H3 was accepted in the 2012 survey.

The 2015 survey results revealed regional differences in the magnitude of effect food values had on purchase intent. In Tokyo, social value (β = 0.45) remained the most influential value, the order of the magnitudes of path coefficients of beneficial value (β = 0.33) and safety value (β = −0.25) did not change either. On the other hand, in Osaka, beneficial value had the largest path coefficient (β = 0.40), followed by social value (β = 0.29) and safety value (β = −0.21). Z-test results revealed a significant difference between positive path coefficients of social value and purchase intent, and it was significantly larger in Tokyo than that of Osaka (z = −2.524, *p* < 0.01). No significant regional difference was found in the negative effects of safety value. Therefore, H1 was accepted and H2 was rejected in the 2015 survey. In 2015, the significant regional difference of path coefficient between purchase intent and beneficial value (β_Tokyo_ = 0.33; β_Osaka_ = 0.40) was not detected, thus H3 was accepted.

Overall in Osaka, there were significant reductions in positive path coefficient of social value and negative path coefficient of safety value on purchase intent between in 2012 and 2015. Results of z-test confirmed significant chronological change in path coefficients between purchase intent and social value (z = −2.005, *p* < 0.01), and safety value (z = 2.738, *p* < 0.001).

## 4. Discussion

This study aimed to evaluate the impact of food value on consumer purchase intent by contextualizing the issue in the affected area by the 2011 Great East Japan earthquake and focusing on one specific marine product (i.e., salted salmon). Three categories of food values were identified, i.e., safety value, social value, and beneficial value. Hypotheses were formulated based on the assumption that there might be a regional difference in the effects food value exerts on consumer purchase intent. Surveys were conducted among consumers living in two cities (i.e., Tokyo and Osaka) to determine to what extent food value affected consumer purchase intent. Further analysis was conducted to determine whether a regional difference might persist one year and four years after the disaster (i.e., 2012 and 2015). The major findings of this research are as follows.

### 4.1. Social Value

Results of 2012: The hypothesis H1 states that consumer perception of social value was a greater motivator for purchase intent in Tokyo than in Osaka. In 2012, almost one year after the disaster, consumer perception of social value was found to have the greatest positive impact on purchase intent not only in Tokyo but also in Osaka, with no significant regional differences, and therefore H1 was rejected base on the 2012 data. This suggests that a nationwide awareness of support for the disaster-affected area was effectively enhanced among consumers with shared social identity with victims and in-group favoritism, as assumed by SCT and CRT. This awareness increased their perceived social value of the salmon, which in turn promoted consumer purchase intent. It was also found that social value was a greater purchase intent promotor than beneficial value.

Results of 2015: Almost four years after the disaster, a regional difference was detected in the association between social value and purchase intent. That is, the magnitude of effect of social value on purchase intent was larger in Tokyo than in Osaka, thus verifying H1. In Tokyo, social value still had the greatest positive impact on purchase intent in 2015. In addition, comparison a between the 2012 and 2015 results showed a significant reduction in the effect of social value only with Osaka consumers. This difference can be explained by individual’s previous disaster experience [22]. That is, Tokyo consumers showed consistent solidarity with the victims even four years after the disaster, mainly because they were also affected by big seismic motion, distribution network turmoil and planned blackouts during the 2011 disaster. Regarding social identity sharing and the resultant solidarity, Collins [47] who examined these issues in the context of historical disasters such as 9/11, pointed out that it was a temporary phenomenon which would last a relatively short period of time. It would often reach a peak during two to three months before returning to normal during six to nine months after the disaster. However, in the current study, a significant association between social value and purchase intent stimulated by the disaster still existed after a relatively long period of time (e.g., one year) after the disaster, and lasted even longer in the neighboring area (i.e., Tokyo) afterwards, confirming a regional difference in this association over time. This inconsistent finding can be interpreted with various factors directly or indirectly attributable to the disaster, including psychological solidarity, and the recognition of the long road toward recovering from the disaster.

### 4.2. Safety Value

Results of 2012: The hypothesis H2 states that consumer perception of safety value was a greater suppressor for purchase intent in Osaka than in Tokyo. This hypothesis was verified in the 2012 survey. This result was consistent with previous studies conducted immediately after the disaster [11,12] which documented growing concerns about radioactive contamination expressed by people living in places far away from the disaster-affected areas. Furthermore, it also found that safety value suppressed purchase intent to almost the same extent as beneficial value promoted purchase intent. The implication of this finding is that in post-disaster sales recovery, the same levels of communication efforts are needed to mitigate the negative impact caused by safety value concerns about radioactive contamination as well as to promote beneficial value of the product.

Results of 2015: By contrast, analyses of the 2015 data showed no significant difference in perception of safety value between Tokyo and Osaka. Furthermore, z-test results revealed a significant reduction in negative effect only in Osaka. That is, the negative effect observed in Osaka in 2012 decreased to the same level as Tokyo in 2015 with the passage of time, hence rejecting H2. The implication of this finding partially agrees with that of Aruga [12]. That is, immediately after a nuclear accident occurs, it would be feasible and desirable to draw up a strategy to resume distribution of products from disaster-affected areas in relatively nearby markets. However, the study finding suggests that sales enhancement measures may also be relevant in relatively remote markets as time passes.

### 4.3. Beneficial Value

The hypothesis H3 states that the effect of beneficial value on purchase intent would not differ from one region to the other. H3 was accepted in the cases of Tokyo and Osaka, in 2012 and 2015. This suggested that beneficial value was found to be a value promoting purchase intent, regardless of the geographical distance from the disaster-affected area.

In addition, analyses of the 2015 data revealed a shared tendency in Tokyo and Osaka, indicating that the absolute value of the negative effect of safety value was smaller than that of the positive effect of beneficial value. Dissimilar to the implications generated from the 2012 results, findings of the 2015 survey imply that it would be more effective to focus on communicating beneficial value of the salted salmon products in comparison with the measures (e.g., providing research-informed data) taken against the safety value concerns.

### 4.4. Correlation among Food Values

Moreover, additional findings were generated on the correlations among latent variables representing values of the salted salmon products. The results of factor correlation analysis on the data of all respondent groups revealed relatively large factor correlations between social value and beneficial value, indicating that safety value and social value were independent of each other, and that safety value and beneficial value were independent of each other as well. This suggests that measures to mitigate the negative effect of safety value on purchase intent (e.g., providing research-based information [9,20]) fail to add to the positive effect that beneficial value and social value exerted on purchase intent. This also suggests that measures to promote purchase intent by communicating beneficial value and social value failed to reduce the negative effect that safety value exerted on purchase intent.

### 4.5. Novelty and Limitations

A number of studies so far have looked at consumers’ negative attitudes toward agricultural and livestock products from disaster-affected areas in the context of the Great East Japan earthquake, particularly the Fukushima nuclear plant accident. A novelty of this research, however, is that it is the first attempt to focus on one marine product (i.e., salted salmon products), assessing the positive effect of food value (i.e., social value and beneficial value) on purchase intent on one hand, and the negative effect of food value (i.e., safety value) on purchase intent on the other. It revealed regional and time-related differences in the magnitude of these effects. However, it suffers several limitations due to its scope. Firstly, the salted salmon products in this study originated from Miyagi Prefecture where distribution of marine products had already been resumed as of 2012; that is, the product examined in the study was not actually from Fukushima Prefecture where the nuclear accident took place. Therefore, the study findings needs to be supplemented by future studies on safety value of marine products and its relation to consumer perception and purchase intention, in particular case studies on Fukushima’s marine products, which are associated with higher food safety awareness on part of the consumers. Secondly, since this research targeted only salted salmon products originating from aquaculture in Miyagi, further investigations on other types of food are needed to determine whether there are similar tendencies in the association between food values and purchase intention as suggested by this study. This line of research will advance our understanding of the impact of the Great East Japan Earthquake on consumer perception related to food values. Thirdly, this research addressed the psychological aspects of how food values of fishery products affected consumer purchase intent, with a focus on regional differences; thus, the economic aspects (e.g., market demand and supply trends) of disaster-affected marine products were not examined.

## 5. Conclusions

This research identified the values of food produced in an area affected by natural and technological disasters in the context of the seafood industry, and clarified that effects of food values on consumer purchase intent varied in different spatial and chronological contexts.

With regard to salted salmon products, the present study found that the negative effect of consumer concern about safety value on purchase intent was significantly higher in the relatively distant market than the neighboring market about one year after the disaster. By contrast, consumer perception of the salmon’s social values in contributing to the restoration of the disaster-affected area had the largest impact on purchase intent, the magnitude of which did not differ by the distance from the disaster-affected area. Thus, it would be recommended to focus on sales resumption in neighboring markets and making efforts in prioritizing the dissemination of the product’s social value. As for the magnitude of the negative effect of safety value on purchase intent, it decreased even in the relatively distant market to the same level as the neighboring market four years after the disaster. Therefore, even in the case where the negative effect of safety value on purchase intent is higher in a distant market than in a neighboring one, the passage of time would enable us to enhance sales efforts even in a relatively distant market. While social values promote purchase intent mostly in neighboring markets, it would be worthwhile to transmit the beneficial value of the product (e.g., taste and nutritional value) to relatively distant markets.

Hence, we consider social value is more important than safety and beneficial values in terms of path coefficient, which means that sales promotion with emphasis on social value is more effective than with other values. However, it is necessary to communicate on three values in parallel, in order to fulfill the purpose of promoting consumers’ purchase of seafood products produced in the disaster-affected area. Therefore, this research implies that value transfer strategies may be different according to geographical and temporal diversity.

## Figures and Tables

**Table 1:** Questionnaire on consumer perception and purchase intent of the value of Miyagi salmon.

Construct	Items	Measurement
Taste	Miyagi salmon tastes good	Five-point Likert scale 1: Strongly disagree 2: Disagree 3: Undecided 4: Agree 5: Strongly agree
Price	Miyagi salmon is priced higher than other salted salmon products	
Nutrition	Miyagi salmon is rich in nutrition	
Seasonality	Miyagi salmon is valued as a seasonal product	
Appearance	Miyagi salmon looks good and fresh	
Naturalness *	Farming of Miyagi salmon has a large environmental load because salmon farming seems to need antibiotics	
Convenience *	Miyagi salmon is easy to cook because it is prepared without bones	
Safety 1	I have concerns about the institutions responsible for inspecting Miyagi salmon	
Safety 2	I have concerns about the method of inspection (complete or sampled) on radioactivity of Miyagi salmon	
Social 1	By purchasing Miyagi salmon, we contribute to the reconstruction of disaster-affected areas	
Social 2	By purchasing Miyagi salmon, considerable employment can be created in disaster-affected areas	
Social 3	By purchasing Miyagi salmon, we can protect rural fishery culture (fishery industry, cooking methods) in disaster-affected areas	
Origin *	If there is no difference in the price of salmon between Miyagi and overseas products, I want to buy Miyagi salmon	
Environment *	Harvesting of Miyagi salmon has a large environmental load for nature because it needs huge amounts of feed	
Purchase intent	I want to purchase Miyagi salmon for home consumption	

Note: * indicates questions which were excluded from the 2015 questionnaire.

**Table 2:** Results of factor analysis and correlations among constructs in the 2012 survey for the two groups (*N*_Tokyo_ = 340; *N*_Osaka_ = 319).

		Tokyo			Osaka		
		Beneficial	Social	Safety	Beneficial	Social	Safety
Taste		0.585	0.186	0.019	0.685	−0.008	−0.003
Nutrition		0.733	−0.036	0.076	0.778	0.010	0.062
Seasonality		0.811	−0.042	−0.046	0.840	0.006	−0.029
Appearance		0.758	−0.008	−0.050	0.669	0.132	0.004
Price		0.554	−0.027	0.015	0.652	−0.106	−0.037
Safety 1		0.010	−0.004	0.909	−0.014	0.011	0.982
Safety 2		−0.007	0.002	0.969	0.005	−0.020	0.909
Social 1		−0.007	0.882	0.024	−0.008	0.908	−0.056
Social 2		0.033	0.918	0.004	−0.025	0.969	0.021
Social 3		−0.036	0.908	−0.031	0.012	0.923	0.027
KMO measure of sampling adequacy		0.781			0.785		
Bartlett’s test of sphericity	Approximate chi-squared	1977.771			2225.943		
	*df*	45			45		
	Significant	0.000			0.000		
Cronbach’s alpha		0.817	0.928	0.937	0.847	0.943	0.950
eigenvalue		3.003	3.007	1.841	3.319	3.284	1.847
Correlations	Beneficial	1			1		
	Social	0.458	1		0.488	1	
	Safety	0.158	−0.010	1	0.133	0.020	1

Note: *df* = degree of freedom; items with factor loadings above 0.4 are presented in boxes. KMO = Kaiser–Meyer–Olkin.

**Table 3:** Results of factor analysis and correlations among constructs in the 2015 survey for the two groups *N*_Tokyo_ = 722; *N*_Osaka_ = 472).

		Tokyo			Osaka		
		Beneficial	Social	Safety	Beneficial	Social	Safety
Taste		0.627	0.081	−0.029	0.753	0.046	−0.053
Nutrition		0.730	−0.019	0.085	0.782	−0.007	0.042
Seasonality		0.828	−0.023	−0.091	0.776	0.014	−0.023
Appearance		0.707	0.087	0.031	0.739	0.046	0.035
Price		0.649	−0.079	0.018	0.697	−0.084	−0.007
Safety 1		0.019	0.001	0.912	0.034	−0.009	0.993
Safety 2		−0.010	0.000	0.981	−0.037	0.010	0.893
Social 1		0.021	0.877	−0.011	−0.019	0.871	−0.005
Social 2		−0.022	0.912	0.022	−0.002	0.947	−0.006
Social 3		0.003	0.903	−0.011	0.016	0.924	0.013
KMO measure of sampling adequacy		0.794			0.788		
Bartlett’s test of sphericity	Approximate chi-squared	4396			3180		
	*df*	45			45		
	Significant	0.000			0.000		
Cronbach’s alpha		0.864	0.937	0.940	0.834	0.925	0.945
Eigenvalue		3.422	3.172	1.824	3.242	3.156	1.835
Correlations	Beneficial	1			1		
	Social	0.521	1		0.477	1	
	Safety	0.088	0.006	1	−0.109	−0.005	1

Note: *df* = degree of freedom; items with factor loadings above 0.4 are presented in boxes. KMO = Kaiser–Meyer–Olkin.

**Table 4:** Goodness of fit indices in SEM for the four groups.

		2012		2015	
		Tokyo	Osaka	Tokyo	Osaka
Goodness of fit indices	GFI	0.964	0.954	0.973	0.963
	AGFI	0.942	0.926	0.956	0.941
	RMSEA	0.047	0.059	0.051	0.056

Note: GFI = goodness-of-fit index; AGFI = adjusted goodness-of-fit index; RMSEA = root mean square error approximation; SEM = structural equation modeling.

**Table 5:** Results of multiple-group SEM and goodness of fit indices.

		2012			2015		
		Tokyo	Osaka	z	Tokyo	Osaka	z
Standardized path coefficient (β)	Beneficial value →Purchase intent	0.220	0.314	1.228	0.328	0.403	0.234
	Social value →Purchase intent	0.486	0.405	−0.934	0.453	0.293	−2.524 ***
	Safety value →Purchase intent	−0.201	−0.324	−2.472 ***	−0.246	−0.209	0.903
	Beneficial value ⇔Social value	0.474	0.500	−0.702	0.538	0.491	−0.195
Goodness of fit indices	GFI	0.965					
	AGFI	0.944					
	RMSEA	0.026					

Note: *** significant at *p* < 0.001; GFI = goodness-of-fit index; AGFI = adjusted goodness-of-fit index; RMSEA = root mean square error approximation.
